# Minimally Invasive Percutaneous Plate Osteosynthesis technique combined with locking compression plates guided by C-Arm X-Ray machine in treatment of tibial metaphyseal fractures: Curative effect and postoperative complications

**DOI:** 10.12669/pjms.38.3.4757

**Published:** 2022

**Authors:** Xu Zhang, Bin Liu, Quan Wang, Hongtao Shang

**Affiliations:** 1 Xu Zhang, Department of Orthopedics, Orthopedic Hospital of Sichuan Province, Chengdu 610041, Sichuan, China; 2 Bin Liu, Department of Orthopedics, Harrison International Peace Hospital, Hengshui 053000, Hebei, China; 3 Quan Wang, Department of Orthopedics, Harrison International Peace Hospital, Hengshui 053000, Hebei, China; 4 Hongtao Shang, Department of Orthopedics, Harrison International Peace Hospital, Hengshui 053000, Hebei, China

**Keywords:** C-arm X-ray machine, MIPPO, LCP, Tibial metaphyseal fracture, Curative effect

## Abstract

**Objectives:**

To explore the curative effect of minimally invasive percutaneous plate osteosynthesis (MIPPO) technology combined with locking compression plates (LCP) guided by C-arm X-ray machine in the treatment of tibial metaphyseal fracture and its impact on the surgical indexes and postoperative complications.

**Methods:**

The present study was a retrospective analysis focusing on the clinical data of 104 cases of tibial metaphyseal fracture who were treated surgically in our hospital from February 2019 to February 2021. According to different surgical methods, patients who were treated by using MIPPO technology combined with LCP guided by C-arm X-ray machine were divided into the observation group (n=54), while those who underwent traditional open reduction and internal fixation were classified into the control group (n=50). Further comparison was made on the differences of the curative effect, surgical index (operation time, intraoperative blood loss, incision length, and healing time) and postoperative complications between the two groups. In addition, the differences in ankle function, knee function and quality of life [Medical Outcomes Study 36-item Short-Form Health Survey (MOS SF-36)] were evaluated between the two groups before treatment and 6 months after treatment.

**Results:**

The clinical curative effect and total efficacy of the observation group were better than those of the control group (All p<0.05). The operation time, intraoperative blood loss, incision length, and healing time were lower in the observation group when compared to those of the control group (All p<0.05). The total incidence of postoperative complications was also lower in the observation group than that in the control group (p<0.05). In addition, the scores of ankle function, knee function and MOS SF-36 in both groups were higher after 6 months of treatment than those before treatment; besides, the inter-group comparison showed that the scores of ankle function, knee function and MOS SF-36 in the observation group were higher than those in the control group (All p<0.05).

**Conclusion:**

MIPPO technology combined with LCP guided by C-arm X-ray machine has a significant curative effect on the treatment of tibial metaphyseal fracture. It can significantly improve the surgical index, reduce postoperative complications, and have an obvious effect on postoperative recovery of ankle function, knee joint function and quality of life.

## INTRODUCTION

Tibial metaphyseal fracture is a common type of fracture clinically, which is generally caused by high-energy injury. Severe soft tissue injury, as well as significantly declined ankle function and knee function, are common complications of these patients, which has a great impact on the quality of life of the affected patients.^1^,^2^ Therefore, it is necessary to apply active treatment for patients with tibial metaphyseal fracture, so as to recover the ankle function and knee function in a rapid manner. Surgery is the primary choice for the treatment of tibial metaphyseal fracture patients. The surgical treatment approaches include open reduction and internal fixation, intramedullary fixation system, external fixator, etc.^3^,^4^

In recent decades, with the development of minimally invasive technology, minimally invasive percutaneous plate osteosynthesis (MIPPO) technology combined with locking compression plates (LCP) is gradually applied in the treatment of tibial metaphyseal fractures.^5^ MIPPO technology combined with LCP exhibits advantages of less trauma, fracture microenvironment protection, reduction of necrosis of cells and tissues around the fracture, and improvement of fracture healing.^6^,^7^ In view of the above, this study was carried out to explore the curative effect of MIPPO technology combined with LCP under the guidance of C-arm X-ray machine in the treatment of tibial metaphyseal fracture and its impact on the surgical indexes and postoperative complications.

## METHODS

### Subjects of Study

The present study was a retrospective analysis focusing on the clinical data of 104 cases of tibial metaphyseal fracture who were treated surgically in our hospital from February 2019 to February 2021. According to different surgical methods, patients who were treated by using MIPPO technology combined with LCP guided by C-arm X-ray machine were divided into the observation group (n=54), while those who underwent traditional open reduction and internal fixation were classified into the control group (n=50). There was no significant difference in general clinical data between the two groups (All *p*>0.05; Table-I), suggesting comparability between groups.

**Table-I:** Comparison of clinical data between groups [*x*¯±s, n (%)].

Groups	Gender		Age (years)	Cause of trauma			
	
	Male	Female		Traffic accident	Fall from height	Crush injury	Sprain
Observational group (n=54)	31 (57.41)	23 (42.59)	40.85±8.47	27 (50.00)	15 (27.78)	6 (11.11)	6 (11.11)
Control group (n=50)	26 (52.00)	24 (48.00)	41.56±8.92	23 (46.00)	14 (28.00)	7 (14.00)	6 (12.00)
T/χ^2^ value	0.306		0.416	0.278			
p value	0.580		0.678	0.964			

### Ethical Approval

The study was approved by the Institutional Ethics Committee of Harrison International Peace Hospital, and written informed consent was obtained from all participants.

### Inclusion Criteria:

- Patients who met the diagnostic criteria of tibial metaphyseal fracture^8^;
- Patients with fresh fractures;
- Oatients who aged >18 years old; and
- Patients with complete clinical data.

### Exclusion Criteria:

- Patients with incomplete clinical data;
- Patients with mental disorder and cognitive impairment;
- Patients with malignant tumors;
- Patients with severe heart, liver, kidney and other important organ diseases;
- Critically ill patients;
- Patients with diabetes mellitus;
- Pregnant or lactating women;
- Patients with fractures in other parts;
- Iilliterate patients.

### Traditional Open Reduction and Internal Fixation

Patients were adjusted in their supine position for epidural anesthesia. An arc incision was made by the surgeon at the tibial metaphysis to cut open the skin and subcutaneous tissue layer by layer for full exposure of the fracture end. After that, the hematoma was removed at the fracture end, with attention paid to avoid damage to the blood supply of the fracture end. After the reduction of the fracture end, the end was temporarily fixed with Kirschner wire. The anatomical plate was attached to the fracture end, with the position of the plate adjusted, and then the drill was used to drill and screw. After plate fixation, Kirschner wire was taken out and the incision was sutured layer by layer in the final step.

### MIPPO Technique Combined with LCP Guided by C-Arm X-Ray Machine

Patients were adjusted in their supine position for epidural anesthesia. Before the operation, plaster support was used to fix the fracture end temporarily, and the fracture was pre-reduced in advanced preoperatively. Intraoperatively, C-arm X-ray machine was applied to examine the fracture position of the patients for traction, with the use of percutaneous forceps clip reduction forceps for reduction. The fluoroscopy results obtained were regarded as the basis for the choice of LCP. Furthermore, an incision from the patient’s medial malleolus or knee joint was made by the surgeon, which was about 3-4 cm in length. Periosteal dissector was then utilized by the surgeon to peel off the deep fascia and the soft tissue below the fracture end, and then the appropriate LCP was placed through the formed soft tissue channel. After implantation, the LCP was attached to the surface of the fracture end, and then fixed with screws. Afterwards, the outcome of reduction was identified under the examination of C-arm X-ray machine. The incision was sutured layer by layer after satisfied reduction.

### Postoperative Treatment

Patients in both groups were not bandaged on Day-I after operation, with local placement of drainage tube. Antibiotics were used routinely for one week postoperatively to prevent infection. On second days after operation, the drainage tube was taken out and the incision was bandaged. The dressing was changed every one day for one week. Patients in both groups were followed up regularly for one year.

### Evaluation Criteria of Curative Effect^9^

The curative effect of patients was evaluated according to Johner-Wruhs Criteria.^9^ According to X-ray findings as well as the skin, tissue and joint activity of the affected joint, the scores were graded into excellent (3 points), good (2 points), general (1 point) and poor (0 point). Among them, the excellent and good rate was calculated according to the formula of the rate = (excellent cases+good cases)/total cases×100%.

### Evaluation Criteria of Ankle Function and Knee Function

The American Orthopaedic Foot and Ankle Score (AOFAS) was adopted for the evaluation of patient ankle function.^10^ AOFAS consisted of three dimensions of pain, function and alignment. The total score was 100, and a higher score of patients would indicate a better ankle function. Furthermore, the Lysholm Knee Scale was used to assess the knee function of enrolled patients.^11^ There were eight items in this scale, and a lower score of patients would reveal a worse knee function.

### Evaluation Criteria of Quality of Life^12^

The quality of life of patients was evaluated by Medical Outcomes Study 36-item Short-Form Health Survey (MOS SF-36). MOS SF-36 included five dimensions of physical function, general health, social function, emotional role and mental health, with a full score of 100 for each dimension. A higher score of MOS SF-36 might suggest a better quality of life for the patient.

### Observational Indexes

The observation indexes were the curative effect, surgical index (operation time, intraoperative blood loss, incision length, and healing time) and postoperative complications between the two groups. Besides, the differences in ankle function, knee function and quality of life were evaluated between the two groups before treatment and six months after treatment.

### Statistical Analysis

SPSS22.0 Statistical Software was used for the statistical analysis of this study. The curative effect and postoperative complications were expressed as percentage (%), and compared statistically with *χ*^2^ test, *Fisher*’s exact probability. While surgical index (operation time, intraoperative blood loss, incision length, and healing time), ankle function, knee function, quality of life and other indexes were presented in the form of mean ± standard deviation (±*s*) and statistically analyzed by using *t* test. *P*<0.05 meant that the difference was statistically significant.

## RESULTS

The clinical curative effect and total effective rate of the observation group were better than those of the control group (All *p*<0.05). Table-II.

**Table-II:** Comparison of clinical curative effect in patients between the two groups [n (%)].

Groups	Clinical curative effect				Excellent and good rate

	Excellent	Good	General	Poor	
Observational group (n=54)	32 (59.26)	19 (35.19)	2 (3.70)	1 (1.85)	51 (94.44)
Control group (n=50)	15 (30.00)	25 (50.00)	6 (12.00)	4 (8.00)	40 (80.00)
Z/χ^2^ value	10.470				4.952
p value	0.001				0.026

*Comparison of Surgical Indexes* The operation time, intraoperative blood loss, incision length, and healing time were lower in the observation group when compared to those of the control group (All *p*<0.05). Table-III.The total incidence of postoperative complications was also lower in the observation group than that in the control group (*p*<0.05). Table-IV.

**Table-III:** Comparison of surgical indexes in patients between the two groups (*x*¯±s).

Groups	Operation time (min)	Intraoperative blood loss (ml)	Incision length (cm)	Healing time (week)
Observational group (n=54)	53.85±8.42	70.36±15.63	3.59±0.33	9.54±2.26
Control group (n=50)	81.47±10.94	131.69±31.57	6.85±0.68	13.97±3.63
t value	14.490	12.697	31.463	7.531
p value	0.000	0.000	0.000	0.000

**Table-IV:** Comparison of postoperative complications in patients between the two groups [n (%)].

Groups	Incision infection	Delayed union of fracture	Nonunion of fracture	Total incidence
Observational group (n=54)	1 (1.85)	0 (0.00)	0 (0.00)	1 (1.85)
Control group (n=50)	5 (8.00)	1 (2.00)	1 (2.00)	7 (14.00)
Fisher value	-	-	-	-
p value	-	-	-	0.027

The scores of ankle function and knee function in both groups were higher after six months of treatment than those before treatment; besides, the inter-group comparison showed that the scores of ankle function and knee function in the observation group were higher than those in the control group (All *p*<0.05). Table-V. The scores of MOS SF-36 in both groups were higher after six months of treatment than those before treatment; besides, the inter-group comparison showed that the score of MOS SF-36 in the observation group was higher than those in the control group (All *p*<0.05), Table-VI.

**Table-V:** Comparison of ankle function and knee function in patients of the two groups before and after treatment (*x*¯±s, point).

Groups	Time	AOFAS	Lysholm knee function score
Observational group (n=54)	Before treatment	52.16±12.07	49.34±11.47
	6 months after treatment	86.36±10.58^ab^	84.78±9.52^ab^
Control group (n=50)	Before treatment	53.39±12.21	48.42±11.06
	6 months after treatment	76.52±12.69^b^	77.95±15.54^b^

***Note:*** Inter-group comparison with the control group of the same period, ^a^p<0.05; Intra-group comparison with that before treatment,

b p<0.05.

**Table-VI:** Comparison of MOS SF-36 in patients of the two groups before and after treatment (*x*¯±s, point).

Groups	Time	Somatic function	General health	Social function	Emotional role	Mental health
Observational group (n=54)	Before treatment	61.25±11.42	62.53±11.58	65.59±9.86	67.15±12.07	61.32±9.65
	6 months after treatment	83.36±9.58^ab^	85.46±7.84^ab^	86.63±8.47^ab^	88.71±8.15^ab^	84.15±8.79^ab^
Control group (n=50)	Before treatment	62.39±11.68	61.72±11.09	66.34±9.07	67.68±12.33	60.84±9.45
	6 months after treatment	77.52±14.69^b^	79.06±12.63^b^	81.05±10.94^b^	82.39±15.21^b^	76.86±13.81^b^

***Note:*** Inter-group comparison with the control group of the same period, ^a^p<0.05; Intra-group comparison with that before treatment,

b p<0.05.

## DISCUSSION

Clinically, it is generally recognized that there is a high risk of fracture in the middle and lower tibia owing to the anatomical characteristics of tibia since the upper part of tibia is triangle and the lower part is quadrilateral, resulting in a weak middle part.^13^ On the other hand, the blood vessels and arteries that nourish the tibia are broken while there is a fracture of tibia. Besides, due to a less distribution of peripheral tissue of tibia, it is easy to induce skin necrosis and post-injury infection after fracture, leading to a slow progression of fracture healing.^14^,^15^ Traditional internal fixation muses anatomical plate primarily to fix after open reduction, which, however, are accompanied by disadvantages such as large surgical wound, poor reduction effect, fixation failure, etc.^16^ Significantly, MIPPO is a minimally invasive surgical technique developed in recent decades. Its therapeutic principle is to avoid the exposure of the fracture end to maximize the preservation of the fracture end and its surrounding blood supply for the affected patients, thus providing a good microenvironment for fracture healing.^17^,^18^

According to the results in our study, the clinical curative effect and total effective rate of the observation group were better than those of the control group, indicating that MIPPO technology combined with LCP guided by C-arm X-ray machine is beneficial to improve the surgical efficacy of patients with tibial metaphyseal fracture. It can be explained by the following reasons:

During the surgery using MIPPO technology combined with LCP guided by C-arm X-ray machine, the indirect reduction and percutaneous plate insertion can effectively avoid periosteal dissection, reduce the damage of surgery to blood supply at the fracture site, which is conducive to the postoperative recovery of patients^19^,^20^; There is no additional requirement of external fixation when using MIPPO technology combined with LCP, which may benefit early postoperative rehabilitation training, so as to improve the curative effect of the fracture patients^21^; Compared with traditional anatomical plate, LCP has less pressure on periosteum, with almost no damage to periosteum, which may help to maintain the blood supply at the fracture end, so as to promote the improvement of the curative effect of patients. Simultaneously, in view of the comparison of surgical indicators between groups, the use of MIPPO technology combined with LCP guided by C-arm X-ray machine exhibited a more significant effect on reducing the operation time, intraoperative blood loss, incision length, and healing time. It may be attributed to the following reasons. Firstly, the application of MIPPO technology combined with LCP guided by C-arm X-ray machine can fully avoid periosteal dissection to reduce surgical trauma and thus decrease the risk of iatrogenic bleeding.^22^,^23^ In addition, a larger incision is required during traditional surgery, with the need of peeling the periosteal soft tissue as well, which will cause greater surgical trauma and increase the operation time.

Furthermore, in view of the comparison of postoperative complications between groups, the use of MIPPO technology combined with LCP guided by C-arm X-ray machine might be beneficial to reduce the incidence of complications. As for corresponding reasons, the incision length required by MIPPO combined with LCP is shorter than that of traditional surgery, which is helpful to reduce the risk of incision infection. While the traditional procedure of open reduction may increase the damage of soft tissue, further leading to possible skin necrosis and plate exposure that require additional surgery for repair, which is not conducive to fracture healing.^24^ In our study, after treatment, the ankle function and knee function of the observation group were superior to those of the control group, indicating that MIPPO technology combined with LCP guided by C-arm X-ray machine can promote the recovery of knee and ankle joint function in patients with tibial metaphyseal fracture. The reason may be that the elastic fixation of MIPPO technology combined with LCP allows the micro-movement at the site of fracture, which can stimulate the formation of a large number of callus, promote the secondary healing of fracture, and hence promoting the recovery of ankle function and knee function of patients.^25^ Additionally, as for the quality of life of patients in both groups, it was improved more significantly in the observation group after treatment, which may be related to accelerated postoperative fracture healing, as well as recovery in ankle function and knee joint function in this group.

### Limitations of the study

It includes insufficient sample size of each group, insufficient evaluation indicators, etc., which need to be further studied and improved in the future.

## CONCLUSIONS

MIPPO technology combined with LCP guided by C-arm X-ray machine has a significant curative effect on the treatment of tibial metaphyseal fracture. It can significantly improve the surgical index, reduce postoperative complications, and have an obvious effect on postoperative recovery of ankle function, knee joint function and quality of life.

### Authors’ Contributions:

**XZ and BL** designed this study and prepared this manuscript,and are responsible and accountable for the accuracy or integrity of the work.

**QW** collected and analyzed clinical data.

**HS** significantly revised this manuscript.
